# Metabolic Pathway of Topramezone in Multiple-Resistant Waterhemp (*Amaranthus tuberculatus*) Differs From Naturally Tolerant Maize

**DOI:** 10.3389/fpls.2018.01644

**Published:** 2018-11-21

**Authors:** Anatoli V. Lygin, Shiv S. Kaundun, James A. Morris, Eddie Mcindoe, Andrea R. Hamilton, Dean E. Riechers

**Affiliations:** ^1^Department of Crop Sciences, University of Illinois at Urbana–Champaign, Urbana, IL, United States; ^2^Syngenta, Jealott’s Hill International Research Centre, Bracknell, United Kingdom; ^3^Department of Chemistry, Truman State University, Kirksville, MO, United States

**Keywords:** herbicide metabolism in plants, detoxification, triketone herbicides, pyrazolone, cytochrome P450, oxidative metabolism, herbicide resistance, HPPD inhibitor

## Abstract

Waterhemp [*Amaranthus tuberculatus* (Moq.) Sauer] is a problematic dicot weed in maize, soybean, and cotton production in the United States. Waterhemp has evolved resistance to several commercial herbicides that inhibit the 4-hydroxyphenylpyruvate-dioxygenase (HPPD) enzyme in sensitive dicots, and research to date has shown that HPPD-inhibitor resistance is conferred by rapid oxidative metabolism of the parent compound in resistant populations. Mesotrione and tembotrione (both triketones) have been used exclusively to study HPPD-inhibitor resistance mechanisms in waterhemp and a related species, *A. palmeri* (S. Wats.), but the commercial HPPD inhibitor topramezone (a pyrazolone) has not been investigated from a mechanistic standpoint despite numerous reports of cross-resistance in the field and greenhouse. The first objective of our research was to determine if two multiple herbicide-resistant (MHR) waterhemp populations (named NEB and SIR) metabolize topramezone more rapidly than two HPPD inhibitor-sensitive waterhemp populations (named SEN and ACR). Our second objective was to determine if initial topramezone metabolite(s) detected in MHR waterhemp are qualitatively different than those formed in maize. An excised leaf assay and whole-plant study investigated initial rates of topramezone metabolism (<24 h) and identified topramezone metabolites at 48 hours after treatment (HAT), respectively, in the four waterhemp populations and maize. Results indicated both MHR waterhemp populations metabolized more topramezone than the sensitive (SEN) population at 6 HAT, while only the SIR population metabolized more topramezone than SEN at 24 HAT. Maize metabolized more topramezone than any waterhemp population at each time point examined. LC-MS analysis of topramezone metabolites at 48 HAT showed maize primarily formed desmethyl and benzoic acid metabolites, as expected based on published reports, whereas SIR formed two putative hydroxylated metabolites. Subsequent LC-MS/MS analyses identified both hydroxytopramezone metabolites in SIR as different hydroxylation products of the isoxazole ring, which were also present in maize 48 HAT but at very low levels. These results indicate that SIR initially metabolizes and detoxifies topramezone in a different manner than tolerant maize.

## Introduction

Topramezone is a 4-hydroxyphenylpyruvate dioxygenase (HPPD)-inhibiting herbicide primarily used postemergence (POST) in maize (*Zea mays* L.) for broadleaf and grass weed control (Grossmann and Ehrhardt, 2007; Gitsopoulos et al., 2010). Herbicides that inhibit the HPPD enzyme cause sensitive plants to die by depleting plastoquinone, which in turn leads to depletion of tocopherols, carotenoids, and eventual bleaching of leaf tissues and cell membrane damage (Hess, 2000; Pallett et al., 2001; Ndikuryayo et al., 2017). Maize possesses natural tolerance to topramezone via rapid oxidative metabolism of the parent compound, specifically an *N*-demethylation reaction, which is presumably catalyzed by cytochrome P450 monooxygenase (P450) enzyme activity (Grossmann and Ehrhardt, 2007). Recent field and greenhouse studies reported resistance to several POST HPPD inhibitors in waterhemp (*Amaranthus tuberculatus*) and Palmer amaranth (*A. palmeri*), including topramezone (Hausman et al., 2011, 2016; Oliveira et al., 2017; Heap, 2018), as well as resistance to the photosystem II inhibitor atrazine by distinct metabolic mechanisms (Ma et al., 2013).

**FIGURE 1:**
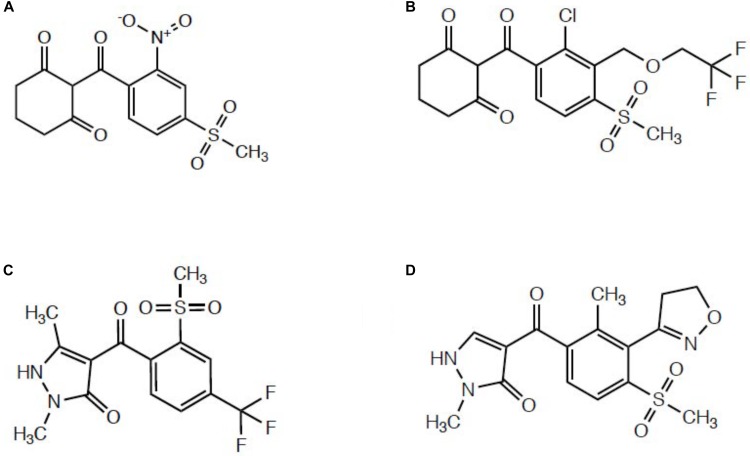
Representative structures from different subclasses of commercial HPPD-inhibiting herbicides. **(A)** Mesotrione, a triketone. **(B)** Tembotrione, a triketone. **(C)** Pyrasulfotole, a pyrazolone. **(D)** Topramezone, a pyrazolone.

Several published reports indicated enhanced oxidative metabolism of either mesotrione (Ma et al., 2013; Kaundun et al., 2017; Nakka et al., 2017) or tembotrione (Küpper et al., 2018) contributes significantly to whole-plant resistance levels relative to HPPD inhibitor-sensitive populations. Since these two herbicides belong to the triketone subfamily of HPPD-inhibiting herbicides (Figure 1) (Lee et al., 1998; Ndikuryayo et al., 2017), it is not surprising that metabolic resistance in *Amaranthus* populations proceeds via 4-hydroxylation of the cyclohexanedione ring, which is the same mechanism underlying maize tolerance and selectivity (Hawkes et al., 2001). Mechanistic research investigating topramezone metabolism has not been reported in multiple herbicide-resistant (MHR) *Amaranthus* populations, yet studying topramezone detoxification in MHR plants is of great interest since topramezone belongs to the pyrazolone subfamily of HPPD inhibitors (Figure 1) (Siddall et al., 2002; Grossmann and Ehrhardt, 2007; Ndikuryayo et al., 2017). It remains to be experimentally determined whether populations resistant to HPPD-inhibiting herbicides mimic maize by detoxifying topramezone by *N*-demethylation (Grossmann and Ehrhardt, 2007) or via ring/alkyl hydroxylation at a liable position, as is the case for mesotrione (Hawkes et al., 2001; Ma et al., 2013).

The two MHR waterhemp populations studied to date (MCR/SIR from Illinois and NEB from Nebraska; both HPPD inhibitor and *s*-triazine resistant) utilized foliar-applied mesotrione to investigate degradation rates and identify metabolites compared with sensitive populations (Ma et al., 2013; Kaundun et al., 2017). However, although both populations also exhibit resistance to POST topramezone (Hausman et al., 2016; Kaundun et al., 2017), only the SIR population had prior exposure to the pyrazolone topramezone in the field (Hausman et al., 2011). These differences in field-use histories of HPPD inhibitors between populations previously led us to speculate that mesotrione and/or tembotrione may have selected for cross-resistance to topramezone via enhanced oxidative metabolism (Kaundun et al., 2017). However, since topramezone does not possess a cyclohexanedione ring as with the triketones (Figure 1), we hypothesized that the same P450(s) catalyzing 4-hydroxylation of the dione ring of mesotrione and tembotrione in HPPD-resistant waterhemp might also catalyze *N*-demethylation of topramezone in MCR/SIR and NEB, similar to the initial detoxification reaction in tolerant maize (Grossmann and Ehrhardt, 2007).

As a result, the objectives of our current research toward further investigations of HPPD-inhibitor resistance mechanisms in waterhemp are two-fold: (1) determine if two MHR waterhemp populations metabolize topramezone faster than two HPPD inhibitor-sensitive populations, and (2) qualitatively determine if initial topramezone metabolite(s) formed in waterhemp are different than those in maize. Our results shed new light on the multigenic, complex inheritance patterns for HPPD-inhibitor resistance (studied with mesotrione only; Huffman et al., 2015; Kohlhase et al., 2018) by demonstrating that MHR waterhemp populations have the potential to evolve complex metabolic mechanisms leading to cross- and/or multiple resistance that might be herbicide-dependent, and may also differ from mechanisms in naturally tolerant cereal crops.

## Materials and Methods

### Plant Materials

The four waterhemp populations investigated in this research are the same as those described by O’Brien et al. (2018). Two are sensitive to HPPD-inhibiting herbicides (SEN and ACR) and the two others (SIR and NEB) are resistant to POST applications of mesotrione, tembotrione, topramezone, and atrazine (Hausman et al., 2011; Kaundun et al., 2017). The SIR population was sampled from the same field site as the MCR population described in Hausman et al. (2011). Hybrid corn (DKC 63-14 RR) was used for comparison with waterhemp.

### Whole-Plant POST Herbicide Dose-Response Study

For conducting dose-response studies with topramezone in the greenhouse, approximately 30 seeds of each waterhemp population were directly sown in 10-cm diameter pots containing a commercial potting medium of sandy-loam soil. For corn, approximately 10 seeds were sown per pot. Each pot (comprising one replicate) was maintained in a greenhouse providing a 16/8 h photoperiod of 180 μmol m^−2^ s^−1^ with day/night temperatures of 24/18°C at constant 65% relative humidity.

When waterhemp plants were 7-cm tall and corn plants reached the 2–3 leaf stage, they were treated with topramezone (Armezon^TM^, BASF Corp., NC, United States) at 0, 0.1, 0.2, 0.39 0.78, 1.56, 3.13, 6.25, 12.5, 25, 50, or 100 g ai ha^−1^ using a track sprayer fitted with a Teejet nozzle calibrated to deliver 200 L ha^−1^. All treatments included Agridex 1% (v/v) (Helena Chemical) as well as ammonium sulfate at 2.5% (w/v) as spray adjuvants. Following herbicide treatments, each pot (one replicate) was arranged within a randomized complete block design and maintained in the greenhouse as described above. Five replicate pots were used per herbicide treatment and population for SEN, SIR, NEB, and corn. Due to limited seed availability only three replicate pots were utilized for ACR. Pots were assessed for visual percent control compared to an untreated control at 21 days after treatment (21 DAT). Percent visual control of maize was zero across all rates of topramezone tested (data not shown) while typical dose responses were generated for each waterhemp population.

### Topramezone Metabolism in Excised Waterhemp and Maize Leaves

Waterhemp seeds were suspended in 0.1 g L^−1^ agar:water solution at 4°C for at least 30 days to enhance germination. Seeds from each waterhemp population were germinated in 12 cm × 12 cm trays with a commercial potting medium (Sun Gro Horticulture, Bellevue, WA, United States) in the greenhouse. Emerged seedlings (2-cm tall) were then transplanted into 80 cm^3^ pots in the greenhouse. When the seedlings were 4-cm tall they were transplanted into 950 cm^3^ pots containing a 3:1:1:1 mixture of potting mix:soil:peat:sand. The soil component contained 3.5% organic matter with a pH of 6.8. Slow-release fertilizer (Osmocote, The Scotts Company, Marysville, OH, United States) was added to this mixture. Corn seeds were planted 2.5-cm deep in the same soil mixture. Plants at a height of 10–12 cm were transferred to a growth chamber for 24-h before conducting herbicide metabolism studies with either excised leaves or whole plants, as described below. Greenhouse and growth chamber (Controlled Environments Limited, Winnipeg, Canada) conditions were maintained at 28/22°C day/night with a 16/8 h photoperiod. Natural sunlight was supplemented with mercury halide lamps, providing a minimum of 500 μmol m^−2^s^−1^ photon flux at plant canopy level in the greenhouse. Light in the growth chamber was provided by incandescent and fluorescent bulbs delivering 550 μmol m^−2^s^−1^ photon flux at plant canopy level.

Excised leaves were prepared according to the protocol of Ma et al. (2015) with minor amendments as described below. On the day of the experiments, the fourth youngest leaves of waterhemp plants (one from each plant) were collected in a container with water, leaf petioles were cut again with razor blade under water and placed in 1.5 mL plastic tubes containing 200 μL of 0.1 M Tris-Cl buffer (pH 7.5) to equilibrate for an hour. Leaves were then transferred to new 1.5 mL tubes containing herbicide incubation solution [150 μM topramezone in 0.1 M Tris-Cl buffer (pH 7.5)]. The youngest corn leaf from 10–12 cm plants was processed as above with waterhemp leaves for comparison. Excised leaves were incubated in the 150 μM topramezone solution for 1 h to allow herbicide uptake, then washed with 0.1 M Tris-Cl buffer (pH 7.5) and placed in 1.5 mL tubes containing 500 μL of one quarter-strength MS salts liquid media. Leaves were harvested at 0 (immediately after the one hr incubation), 2, 5, 11, and 23 h after removal from the topramezone uptake solution [representing 1, 3, 6, 12, and 24 h after treatment (HAT)] by briefly rinsing in deionized water and drying with tissue paper. Tissue fresh weights were recorded, then leaves were placed in 2 mL tubes with screw caps and immediately frozen in liquid nitrogen. Frozen leaves were stored at −80°C until further analysis. Each plant population x time point consisted of four replicates (i.e., excised leaves from different plants) and three independent experiments were conducted. Only the relative concentrations of topramezone remaining at 6 and 24 HAT are reported, which were normalized to the average of the 1-h concentrations (separately for each population and repeated experiment) for the purposes of statistical analysis, as described in detail below.

Leaf samples were freeze-dried with a FlexyDry MP (FTS Systems, Stone Ridge, NY, United States) and ground to a powder with glass beads with a tissue grinder FastPrep FT120, (Savant Instruments Inc., Holbrook, NY, United States). The powder was extracted twice with 80% methanol (1 mL each time) on a rotary shaker at 23°C. The first extraction occurred overnight (at least 16 h) and the second extraction was for 4 h. After shaking, samples were centrifuged at 12000 × *g* for 10 min. Supernatants from the first and second extractions were combined and pellets resulting from each centrifugation step were discarded.

### Sample Preparation and HPLC Analysis

For analysis of topramezone and its metabolites via reverse-phase (RP)-HPLC, a published protocol utilizing UPLC-MS/MS for detecting topramezone in soil, water and plant samples (Li et al., 2011) was modified and optimized for compatibility with waterhemp and maize leaves. Briefly, a 1 mL aliquot of plant extract was placed in a 1.5 mL plastic tube along with 100 μL of 100 μM pyrasulfotole in methanol as an internal standard. Organic solvent was evaporated to incipient dryness with a rotary evaporator (SpeedVac, Farmingdale, NY, United States) and 0.5 mL of 1N HCl (saturated with NaCl) was added followed by 1 mL of methylene chloride. Residue remaining after evaporation was re-dissolved by vortexing and samples were centrifuged at 12000 × *g* for 10 min. An aliquot (800 μL) of the lower methylene chloride layer was removed and placed in a new 1.5 mL tube, then 300 μL of 0.05% NH_4_OH was added and the sample was vortexed to extract topramezone and pyrasulfotole. Samples were centrifuged at 10000 × *g* for 10 min and the upper aqueous layer was carefully collected, stored overnight at 4°C, and subsequently used for RP-HPLC analysis as described below.

The HPLC system consisted of a Waters Alliance separations module (model 2695) equipped with a Waters 996 photodiode array (PDA) detector. Absorbances from 200–400 nm were initially measured with the PDA and 312 nm was selected to quantify parent topramezone and pyrasulfotole, the internal standard. Topramezone was resolved with a Brownlee SPP HPLC column (C_18_, particle size 2.7 μm, 4.6 mm × 100 mm; PerkinElmer). RP-HPLC was performed with binary mobile phases consisting of 5 mM ammonium formate in water:methanol (90:10) as mobile phase A and 5 mM ammonium formate in methanol:water (90:10) as mobile phase B at 30°C and a flow rate of 0.5 mL min^−1^. Samples were loaded in an injection volume of 20 μL and analytes eluted with a gradient of 0–10% B in 10 min, 10–20% B in 5 min, 20–95% B in 4 min, and 95% B for 3 min (isocratic) to wash the column before returning to 0% B for 7 min to re-equilibrate the column prior to analyzing the next sample. For calculation of relative topramezone concentrations, a calibration curve was generated based on the ratio of topramezone to pyrasulfotole peak areas. The calibration curve had an *R*^2^ value of 0.99.

### Topramezone Metabolism in Waterhemp and Maize Plants

The third- and fourth-youngest leaves from waterhemp plants (or youngest leaf from maize plants) were treated with 1.5 mM topramezone [in 0.1 M Tris-Cl buffer (pH 7.5) containing 0.1% (v/v) Tween 20]. Each treated leaf received a total of 20 μL of 1.5 mM topramezone solution applied as ∼0.3 μL droplets with a Hamilton glass syringe. The total amount of topramezone applied corresponded with the amount of topramezone supplied in the incubation solution for the excised leaf experiment. At 24 and 48 HAT, treated leaves (two from each plant) were harvested (including the petioles), washed in 20% methanol to remove unabsorbed topramezone, fresh weights recorded, and leaves frozen in liquid nitrogen. Leaves were stored at −80°C until extraction and further analysis. Two independent experiments were conducted with either two or three replicates of each population × time point after treatment.

Treated leaves were pulverized in liquid nitrogen with a mortar and pestle, and topramezone and its metabolites were extracted with 80% methanol twice (5 mL each time) on a rotary shaker at 23°C. The first extraction occurred overnight (at least 16 h) and the second extraction was for 4 h. After shaking, samples were centrifuged at 12000 × *g* for 10 min. Supernatants from the first and second extractions were combined and stored at 4°C before HPLC analysis, and pellets from each centrifugation step were discarded. The internal standard (pyrasulfotole) was added to each experimental sample, concentrated, re-dissolved and partitioned as previously described. The upper (aqueous) layer was discarded and the lower (organic) layer was carefully collected. A silica solid-phase extraction (SPE) column (500 mg/3 mL loading capacity) was conditioned with 3 mL methylene chloride and the sample was applied to the SPE column, then sequentially washed with 3 mL of methylene chloride followed by 2 mL of methylene chloride:ethyl acetate (1:3) to remove phenolic acids, chlorophylls and pigments. Analytes were eluted from the column with 3 mL of 100% methanol. Methanol was removed with a rotary evaporator, and the flask was washed twice with methanol (0.5 mL each time). The solution was placed in a 1.5 mL plastic tube and methanol removed under a stream of nitrogen gas to dryness. The residue was re-dissolved in 300 μL of methanol, samples were centrifuged at 12,000 × *g* for 10 min, stored overnight at 4°C, and subsequently used for low and high resolution LC-MS analysis as described below. Relative concentrations of topramezone metabolites were determined as described previously for parent topramezone since authentic metabolite standards were either not known or commercially available.

### LC-MS Analyses

Samples for low-resolution LC-MS were analyzed with an Agilent LC-MS (1100 HPLC with XCT Plus Trap mass spectrometer) in the Metabolomics Laboratory of the Roy J. Carver Biotechnology Center, University of Illinois at Urbana-Champaign. LC separation was performed with the same column, mobile phases, and gradient conditions as described above for RP-HPLC analysis of excised leaf extracts, except the flow rate was 0.4 mL min^−1^. The autosampler was set to 15°C and the injection volume was 10 μL. Mass spectra were acquired under negative electrospray ionization (ESI) with dry temperature of 350°C, dry gas flow of 8.5 L min^−1^, and nebulizer gas was set to 35 psi. Mass scan range was 120–900 *m/z*. For MS/MS detection, *m*/*z* 362 was selected as the precursor ion.

Samples for high-resolution LC-MS were analyzed using a Dionex Ultimate 3000 series HPLC system (Thermo, Germering, Germany) and Q-Exactive MS system (Thermo, Bremen, Germany) in the Metabolomics Laboratory of the Roy J. Carver Biotechnology Center, University of Illinois at Urbana-Champaign. Software Xcalibur version 3.0.63 was used for data acquisition and analysis. LC separation was performed with the same column described previously but with different mobile phases and separation conditions. The binary mobile phases consisted of 0.1% formic acid in water as mobile phase A or 0.1% formic acid in acetonitrile as mobile phase B. The flow rate was 0.5 mL min^−1^ and a linear binary gradient was utilized for analyte elution as follows: 0% B for 1 min; 0–60% B in 14 min; 60–100% B in 4 min, 100% B isocratic for 3 min, then the column was returned to 0% B for 8 min before loading the next sample. The autosampler was set to 10°C and the injection volume was 10 μL. Mass spectra were acquired under both positive (sheath gas flow rate, 50; aux gas flow rate: 13; sweep gas flow rate, 3; spray voltage, 3.5 kV; capillary temp, 263°C; aux gas heater temp, 425°C) and negative ESI (sheath gas flow rate, 50; aux gas flow rate, 13; sweep gas flow rate, 3; spray voltage, −2.5 kV; capillary temp, 263°C; aux gas heater temp, 425°C). The full scan mass spectrum resolution was set to 70,000 with the scan range of *m/z* 50 ∼ *m/z* 750, and the AGC target was 1E6 with a maximum injection time of 200 ms. For MS/MS scanning the mass spectrum resolution was set to 17,500. AGC target was 5E4 with a maximum injection time of 50 ms. Loop count was 2 and the isolation window was 1.0 *m/z* with NCE of 25 and 30 eV.

### Statistical Analyses

Whole plant dose-response data for the waterhemp populations were analyzed by straight line regression analysis of logit-transformed visual percent weed control on the logarithm of the rate applied, with the slope of the fitted regression lines being identical for each of the populations (Streibig and Kudsk, 1993). GR_50_s and 95% confidence limits for each population were estimated from the fitted lines. Resistance indices relative to SEN were estimated as the ratio of the respective GR_50_s.

Relative concentrations of topramezone from both the excised leaf and whole plant studies were analyzed by analysis of variance using the linear model:

(1) $$y_{\mathrm{ijkl}}=\mu +\beta_{i}+\pi_{j}+\tau_{k}+{\left(\pi \tau \right)}_{\mathrm{jk}}+\varepsilon_{\mathrm{ijkl}}$$

where *y*_ijkl_ denotes the measured (relative) concentration in replicate l of experiment *i* for population *j* at time *k*, μ is the overall true mean response, β_i_ is the effect of experiment 1, π_j_ is the effect of population *j*, τ_k_ is the effect of time *k*, (πτ)_jk_ is the true effect of the population × time interaction and ε_ijkl_ is the random ‘error’ associated with each individual response. Populations were compared separately at each time point using *t*-tests (α = 0.05) based on the error variance from this model.

## Results

### Whole-Plant Dose-Responses to Topramezone Applied POST in the Greenhouse

**FIGURE 2:**
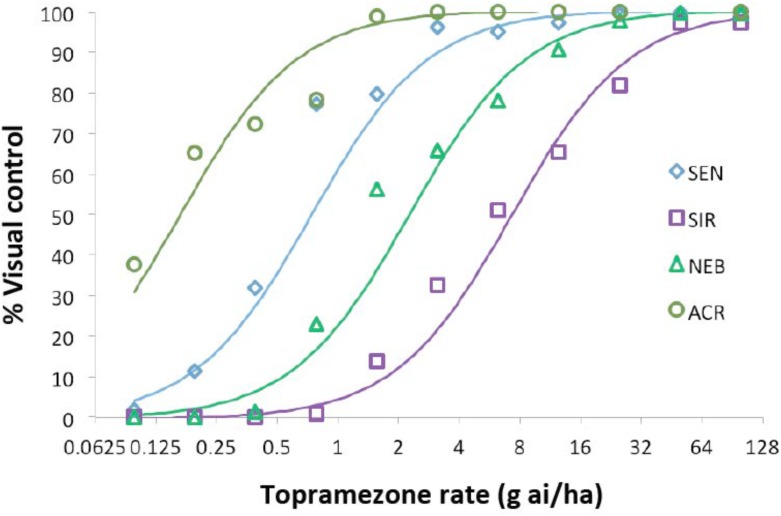
Dose-response analysis of topramezone in four waterhemp populations in the greenhouse. Waterhemp plants (7-cm) from each population were treated with various rates of topramezone, including Agridex at 1% (v/v) (Helena Chemical) and ammonium sulfate at 2.5% (w/v) as spray adjuvants. Plants were assessed for visual percent control compared to an untreated control for each corresponding population at 21 days after treatment. Dose-response curves were generated as described in Section “Materials and Methods” and used to determine the 50% growth reduction and resistance index values listed in Table 1. SIR and NEB are HPPD inhibitor-resistant populations while ACR and SEN are sensitive to HPPD inhibitors (O’Brien et al., 2018).

Four waterhemp populations were subjected to dose-response analysis with topramezone in the greenhouse. Two are sensitive to foliar HPPD-inhibiting herbicides (SEN and ACR) and two (SIR and NEB) are resistant (Hausman et al., 2011; Kaundun et al., 2017; O’Brien et al., 2018). Maize hybrid DKC 63-14 RR was also included for comparison, but data are not shown since this hybrid did not exhibit visual injury symptoms at any topramezone rate tested (24 g ha^−1^ is a field-use rate in maize). SEN, ACR, and NEB were completely controlled at 25 g ha^−1^, while the SIR population exhibited an approximate level of control of 20% (Figure 2). At the lower rates of topramezone examined, ACR was the most sensitive population and NEB was less resistant than SIR. Although both SEN and ACR are sensitive to HPPD-inhibiting herbicides (O’Brien et al., 2018), only the SEN population was utilized to generate resistance indices (RIs) for NEB and SIR (Table 1). Each population displayed GR_50_ values well below the field-use rate of topramezone in maize, ranging from 7.3 g ha^−1^ for SIR to 0.2 g ha^−1^ for ACR (Table 1). In relation to the SEN population, calculated RIs were 9.9 for SIR and 3.1 for NEB.

**Table 1:** Quantitative dose-response analysis of four waterhemp populations based on topramezone rates that cause 50% reductions in plant growth (GR_50_) and resulting resistance indices.

Waterhemp population	GR_50_ values^a,b^ g ai ha^−1^	Resistance index^c^ (relative to SEN)
SEN	0.74 (0.56–0.98)	1.0
SIR	7.34 (5.49–9.77)	9.86 (6.66–14.76)
NEB	2.28 (1.69–3.04)	3.06 (2.06–4.55)
ACR	0.17 (0.11–0.24)	0.22 (0.14–0.35)

### Time-Course Analysis of Topramezone Metabolism in Excised Leaves From Four Waterhemp Populations and Maize

**FIGURE 3:**
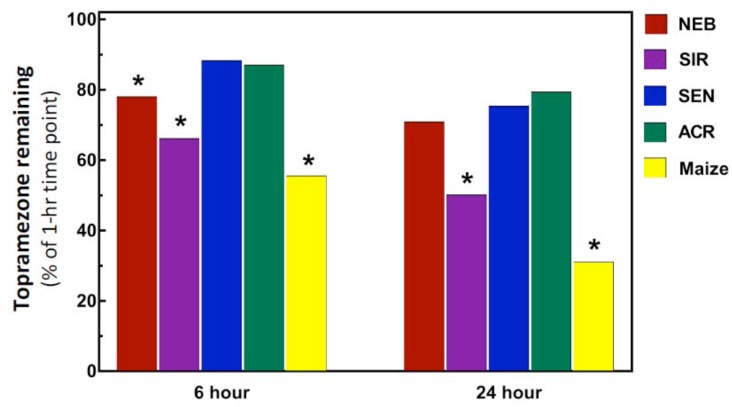
Metabolism of topramezone in four waterhemp populations and maize 6 and 24 hours after treatment (HAT) using an excised leaf assay. Waterhemp and maize seedlings (10–12 cm tall) were grown in the greenhouse and transferred to a growth chamber 24-h before conducting the excised leaf assays, as described previously for mesotrione by Ma et al. (2013, 2015). Excised leaves were incubated for 1-h in a 150 μM topramezone solution, then either harvested immediately or transferred to a dilute MS salts solution for the remainder of the time-course study. Relative concentrations of topramezone remaining in each excised leaf at 6 and 24 HAT (normalized to the average of the 1-h concentrations per population) are plotted on the *Y*-axis, which were determined with reverse-phase HPLC using pyrasulfotole as an internal standard as described in Section “Materials and Methods.” Treatment means significantly different (α = 0.05) than the SEN population mean are marked with asterisks.

Previous studies of initial mesotrione metabolism rates using excised leaves with the HPPD-resistant MCR population (sampled from the same field site as the SIR population) determined rapid mesotrione metabolism within the initial 24 HAT, and a median 50% time for herbicide degradation (DT_50_) of 12-h was calculated for MCR (Ma et al., 2013). Interestingly, the DT_50_ calculated for maize (11.9-h) was almost identical to MCR. By contrast, the SIR population in the current research barely reached 50% topramezone degradation after 24-h while maize displayed a typical degradation curve expected during the time-course analysis (data not shown), achieving approximately 70% topramezone degradation at 24 HAT. As a result of the relatively slower rates of topramezone metabolism in each waterhemp population, only topramezone levels quantified from each population at 6 and 24 HAT are shown in Figure 3.

As expected, topramezone levels in the two HPPD inhibitor-sensitive populations (SEN and ACR) were relatively high, ranging from approximately 80–90% at 6 and 24 HAT (Figure 3). Since SEN and ACR were not different at either time point, only SEN was used as the sensitive population for statistical comparisons with SIR, NEB, and maize. Significant reductions in topramezone were determined for SIR, NEB, and maize at 6 HAT relative to SEN, while only SIR and maize displayed significant reductions at 24 HAT (Figure 3). Lower levels of topramezone in SIR at both time points is consistent with the dose-response analyses (Table 1 and Figure 2), indicating that rapid metabolism contributes to whole-plant resistance to topramezone in SIR. A significant reduction in topramezone levels in NEB at 6 HAT but not at 24 HAT is consistent with the intermediate level of whole-plant resistance to topramezone (relative to SIR and SEN) reported in Table 1.

### Quantification of Topramezone and Its Metabolites Formed 48 HAT in Treated Leaves of Waterhemp and Maize Whole Plants

Whole-plant studies were conducted to corroborate results from the initial experiments with excised leaves (Figure 3) and to further investigate metabolism in treated leaves at later time points after topramezone application (both 24 and 48 HAT), as well as attempt to identify the nature of metabolite(s) formed in MHR waterhemp leaves. Topramezone levels in treated leaves did not differ significantly among waterhemp populations and maize at either time point (*P* = 0.30 for the overall population effect averaged across time; *P* = 0.36 for the population × time interaction), although the effect of time on topramezone metabolism was significant (P = <0.0001; data not shown). These results are in contrast with results determined at 24 HAT in the excised leaf study (Figure 3), where SIR and maize leaves contained less topramezone than ACR, SEN, and NEB. However, since only the treated leaves of whole plants were analyzed at 24 and 48 HAT in this study, basipetal or acropetal topramezone translocation out of the treated leaves to meristematic regions cannot be accounted for. The unavailability of radiolabeled topramezone precluded our ability to examine translocation directly.

**FIGURE 4:**
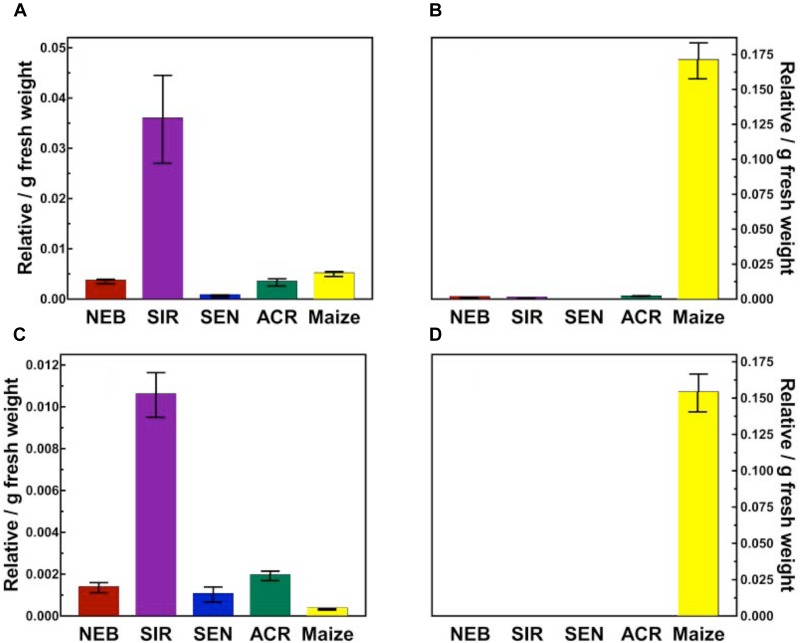
Quantification of topramezone metabolites in treated leaves from four waterhemp populations and maize 48 HAT using whole plants. The third- and fourth-youngest leaves from waterhemp and maize plants (10–12 cm tall) were treated with 60 × 0.33 μL droplets (20 μL total) of a 1.5 mM topramezone solution, including 0.1% (v/v) Tween 20 as a leaf wetting agent, using a glass syringe. Only the treated leaves were harvested from each plant at 24 and 48 HAT (only 48 HAT data are shown), extracted and partially purified by SPE chromatography, then analyzed by LC-MS (low resolution) as described in Section “Materials and Methods.” Relative concentrations of each topramezone metabolite extracted from the treated leaves are plotted on the *Y*-axis (authentic standards were not available), using pyrasulfotole as an internal standard as described in Section “Materials and Methods.” **(A)** Putative hydroxytopramezone-1. **(B)** Desmethyl-topramezone. **(C)** Putative hydroxytopramezone-2. **(D)** Putative benzoic acid metabolite of topramezone (only detected in maize). Vertical bars represent the standard error of the treatment mean.

By contrast, lower amounts of topramezone remaining in excised leaves of SIR and maize at 24 HAT (Figure 3) is supported by the greater abundance of several topramezone metabolites in SIR and maize described below and shown in Figure 4. Initial topramezone metabolism via *N*-demethylation was reported in maize (Grossmann and Ehrhardt, 2007) and the long-term metabolic fate of topramezone has been determined in maize, wheat, and mustard greens (United States Environmental Protection Agency, 2005), but topramezone metabolism has not been reported in weedy species to date. Since radiolabeled topramezone and authentic metabolite standards were not available for our research, relative metabolite quantification and identification was determined via LC-MS using unlabeled topramezone. The pattern of metabolite abundances was not different between 24 and 48 HAT among waterhemp populations and maize treated leaves (data not shown), so only metabolites quantified and identified at 48 HAT are shown in Figure 4.

**FIGURE 5:**
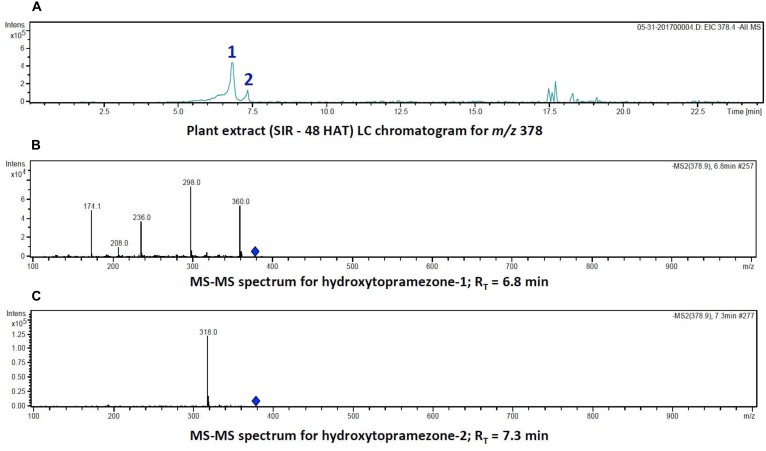
Identification of hydroxytopramezone-1 and hydroxytopramezone-2 metabolites formed in treated leaves of multiple herbicide-resistant waterhemp (SIR) 48 HAT using low-resolution LC-MS. The third- and fourth-youngest leaves from waterhemp and maize plants (10–12 cm tall) were treated with 60 × 0.33 μL droplets (20 μL total) of a 1.5 mM topramezone solution, including 0.1% (v/v) Tween 20 as a leaf wetting agent, using a glass syringe. Only the treated leaves were harvested from each plant (48 HAT), extracted and partially purified by SPE chromatography, then analyzed by LC-MS (low resolution) as described in Section “Materials and Methods.” **(A)** LC analysis of compounds with *m/z* of 378 (M + 16). **(B)** LC-MS/MS spectrum of putative hydroxytopramezone-1 (*R*_T_ = 6.8 min in **A**). **(C)** LC-MS/MS spectrum of putative hydroxytopramezone-2 (*R*_T_ = 7.3 min in **A**).

Two major metabolites were identified and quantified in maize: *N*-demethylated (desmethyl) topramezone as previously reported (Grossmann and Ehrhardt, 2007) and a benzoic acid derivative presumably formed following cleavage of topramezone, which has also been reported previously in maize, wheat, and mustard greens (United States Environmental Protection Agency, 2005). The benzoic acid metabolite was not detected in any waterhemp samples while minor levels of desmethyl-topramezone were detected in NEB, SIR, and ACR (Figure 4). Interestingly, two different putative hydroxylated forms of topramezone (hydroxytopramezone-1 and hydroxytopramezone-2) were identified and quantified in each waterhemp population and maize (Figures 4, 5); in particular, hydroxytopramezone-1 was more abundant in SIR treated leaves relative to other populations.

### Identification and Structural Analysis of Topramezone Metabolites Formed in MHR Waterhemp (SIR Population) and Maize 48 HAT

LC analysis of SIR extracts at 48 HAT showed that the two putative hydroxylated compounds derived from parent topramezone (*m/z* of 378) had similar retention times in the gradient utilized for metabolite separation. The compound eluting first (*R*_T_ = 6.8 min) was tentatively labeled hydroxytopramezone-1 while the compound eluting later (*R*_T_ = 7.3 min) was labeled hydroxytopramezone-2 (Figure 5A). Subsequent LC-MS analysis (with relatively lower resolution; see high resolution below) of each compound revealed that hydroxytopramezone-1 displayed a distinctive fragmentation pattern yielding several informative ions [M – H]^−^ to assist in determining its structure (Figure 5B), while the fragmentation pattern of hydroxytopramezone-2 primarily yielded a major ion at *m/z* of 318 with limited further fragmentation (Figure 5C). As a result, an additional LC-MS/MS analysis was performed with an instrument possessing higher resolution capability to provide additional structural information for metabolite identification.

**FIGURE 6:**
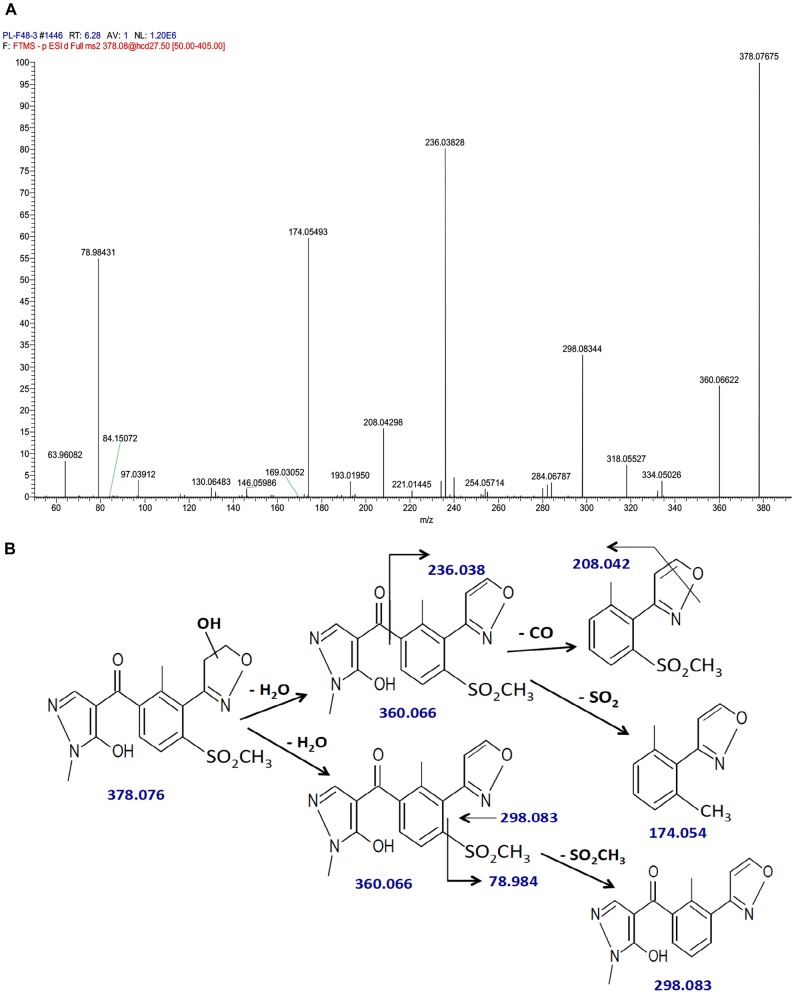
Identification of hydroxytopramezone-1 metabolite formed in treated leaves of multiple herbicide-resistant waterhemp (SIR) 48 HAT using high-resolution LC-MS. The third- and fourth-youngest leaves from waterhemp and maize plants (10–12 cm tall) were treated with 60 × 0.33 μL droplets (20 μL total) of a 1.5 mM topramezone solution, including 0.1% (v/v) Tween 20 as a leaf wetting agent, using a glass syringe. Only the treated leaves were harvested from each plant (48 HAT), extracted and partially purified by SPE chromatography, then analyzed by LC-MS (high resolution) as described in Section “Materials and Methods.” Proposed fragment ions following the loss of –CO (Bouchoux and Hoppilliard, 1981), –SO_2_CH_3_ and intramolecular SO_2_ elimination (Chahboune and Sarakha, 2018) are supported by previous research investigating the fragmentation of isoxazole and mesotrione, respectively. **(A)** LC-MS/MS spectrum of hydroxytopramezone-1. **(B)** LC-MS/MS fragmentation pattern and proposed daughter ion structures.

The fragmentation pattern of hydroxytopramezone-1 with lower resolution LC-MS/MS had yielded ions at *m/z* of 360, 298, 236, 208, and 174.1 (Figure 5B), which were present along with several additional ions at *m/z* of 208.042 and 78.984 using higher resolution LC-MS/MS (Figure 6A). The fragmentation pattern shown in Figure 6B is proposed to account for each major *m/z* peak in Figures 5B, 6A. If the molecular ion at *m/z* of 378.076 represents a hydroxylation of the isoxazoline ring, then loss of a water molecule leads to the fragment at *m/z* of 360.066. In the lower path shown for the loss of water from hydroxytopramezone-1 (Figure 6B), subsequent loss of the sulfone-methyl group [M – SO_2_CH_3_]^−^ (corresponding *m/z* of 78.984) leads to the fragment ion at *m/z* of 298.083 (Figure 6B). In the upper pathway shown for loss of water from hydroxytopramezone-1 (Figure 6B), loss of the pyrazolyl ring and carbonyl group leads to the fragment ion at *m/z* of 236.038 while the corresponding loss of carbon monoxide [M – CO]^−^ from the isoxazole ring (Bouchoux and Hoppilliard, 1981) leads to the ion at *m/z* of 208.042. Alternatively, intramolecular sulfur dioxide elimination leads to the fragment ion at *m/z* of 174.054 (Figure 6B), which was previously reported when analyzing photochemical degradation products of mesotrione (Chahboune and Sarakha, 2018).

**FIGURE 7:**
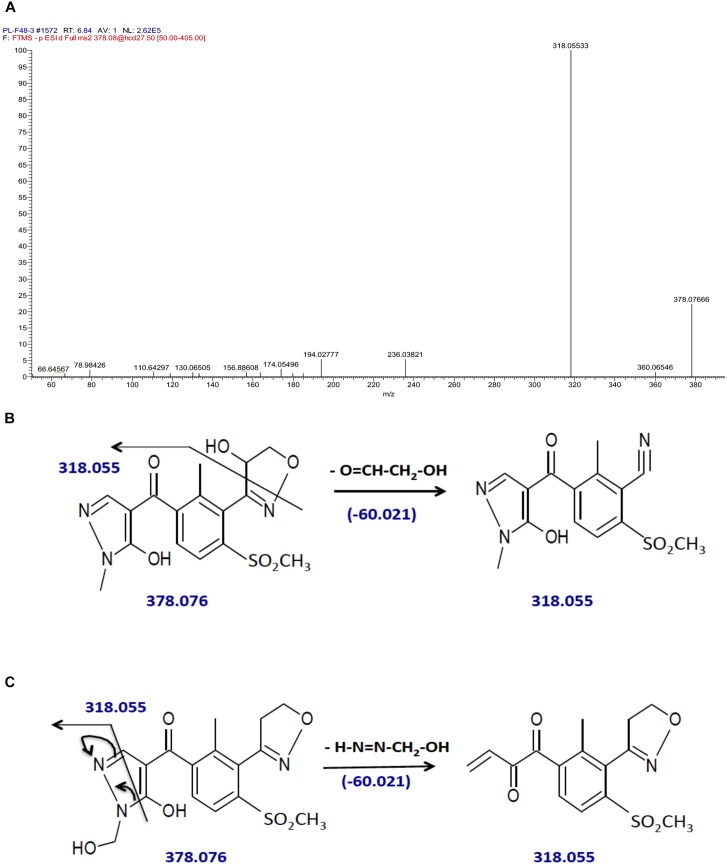
Identification of hydroxytopramezone-2 metabolite formed in treated leaves of multiple herbicide-resistant waterhemp (SIR) 48 HAT using high-resolution LC-MS. The third- and fourth-youngest leaves from waterhemp and maize plants (10–12 cm tall) were treated with 60 × 0.33 μL droplets (20 μL total) of a 1.5 mM topramezone solution, including 0.1% (v/v) Tween 20 as a leaf wetting agent, using a glass syringe. Only the treated leaves were harvested from each plant (48 HAT), extracted and partially purified by SPE chromatography, then analyzed by LC-MS (high resolution) as described in Section “Materials and Methods.” **(A)** LC-MS/MS spectrum of hydroxytopramezone-2, indicating the stable fragment ion with *m/z* of 318.055. **(B)** LC-MS/MS fragmentation pattern and proposed daughter ion structures derived from the putative isoxazole ring hydroxylation metabolite. **(C)** LC-MS/MS fragmentation pattern and proposed daughter ion structures derived from the putative hemi-aminal (i.e., *N-*alkyl hydroxylation) metabolite.

By contrast, hydroxytopramezone-2 yielded only one major fragment ion at *m/z* of 318 (Figure 5C) and 318.055 (Figure 7A) via low and high-resolution LC-MS/MS, respectively, which was also present as a minor fragment ion in the hydroxytopramezone-1 high-resolution spectrum (Figure 6A). The lack of further fragmentation indicates this fragment ion is unusually stable during the LC-MS/MS conditions employed, which is supported by the highly conjugated structures proposed in Figures 7B,C. In either scenario, the daughter ion at *m/z* of 318.055 would result from loss of an exact mass of 60.021 from the parent ion at *m/z* of 378.076. Based on the relative high stability of hydroxytopramezone-2 compared to hydroxytopramezone-1 during our LC-MS/MS conditions, we propose that hydroxylation occurs β to the oxygen in the isoxazoline ring in hydroxytopramezone-2 (Figure 7B; leading to the stable fragment ion at *m/z* of 318.055) whereas hydroxylation occurs α to the oxygen in the isoxazoline ring (thus relatively more electrophilic carbon) in hydroxytopramezone-1 (Figure 6B). However, the existence of the putative hemi-aminal (*N-*alkyl hydroxylation) metabolite (Figure 7C) cannot be excluded at this point without further structural analyses and information.

While alkyl hydroxylation of organic substrates by P450 enzymes is a common reaction (Siminszky, 2006; Mizutani and Ohta, 2010; Urlacher, 2012), it is relatively uncommon for the intermediate hydroxylation product that occurs during heteroatom release (e.g., *O,N*-dealkylation reactions) to accumulate without proceeding further to loss of formaldehyde (Kreuz et al., 1996). For example, the presence of desmethyl-topramezone as a major metabolite in maize leaves (Figure 5) is consistent with P450-catalyzed *N*-demethylation of the pyrazole ring (Grossmann and Ehrhardt, 2007). Formation of the putative hemi-aminal metabolite of topramezone in SIR leaves is thus biochemically less favorable than hydroxylation of the isoxazoline ring at either position (esp. in Figure 7B), but as mentioned previously the hemi-aminal metabolite cannot be excluded without utilizing additional structural analyses such as ^1^H-^13^C-HSQC NMR.

## Discussion

Dose-response analysis demonstrated that the SIR population is more resistant to POST topramezone than the NEB population, which is supported by enhanced topramezone metabolism (Figure 3) and metabolite formation (Figure 4). The greater fold-resistance of SIR may be related to prior usage of topramezone to control the SIR population (along with mesotrione and tembotrione; Hausman et al., 2011), in contrast with NEB (Kaundun et al., 2017), and is also consistent with higher fold-resistance levels of SIR to mesotrione and isoxaflutole applied POST relative to NEB (O’Brien et al., 2018). Moreover, NEB was never pressured with topramezone in the field yet significant levels of resistance were observed, suggesting that the gene(s) selected by mesotrione and/or tembotrione confer cross-resistance to topramezone in the NEB population (Kaundun et al., 2017).

One particularly troublesome aspect of metabolism-based resistance in weeds (Yu and Powles, 2014) is the potential for developing cross-resistance to herbicides from unrelated site-of-action families (Preston, 2004). In the case of HPPD-inhibitor resistance in waterhemp, it is not yet known precisely how many genes govern multigenic resistance (Huffman et al., 2015; Kohlhase et al., 2018), or if one or several P450s contribute to resistance to mesotrione, tembotrione, topramezone, and isoxaflutole (Ma et al., 2013; Kaundun et al., 2017; Nakka et al., 2017; Küpper et al., 2018; O’Brien et al., 2018). In most reported cases of metabolism-based resistance in weeds, mechanisms for metabolic detoxification appear to mimic natural mechanisms for tolerance in crops, leading to the formation of identical metabolite(s) between resistant weed populations and tolerant crops (Holtum et al., 1991; Ma et al., 2013; Yu et al., 2013). Although the precise molecular mechanisms behind enhanced herbicide metabolism in resistant weeds remain unknown, a prevailing theory is that constitutively expressed genes encoding detoxification enzymes [such as glutathione *S*-transferases (GSTs) or P450s] are expressed at higher levels in foliar tissues (Yasuor et al., 2010; Iwakami et al., 2014; Evans et al., 2017; Dyer, 2018). Alternatively, enhanced GST activity (with atrazine as substrate) resulting from an increase in *V*_max_ (Anderson and Gronwald, 1991) and *k*_cat_ (Plaisance and Gronwald, 1999) was documented in metabolic atrazine-resistant *Abutilon theophrasti*. Our results demonstrate that resistant weed populations possess the potential to metabolize herbicide substrates in a different manner than tolerant crops, which further complicates studies aimed at unraveling biochemical and genetic mechanisms that confer metabolism-based weed resistance.

**FIGURE 8:**
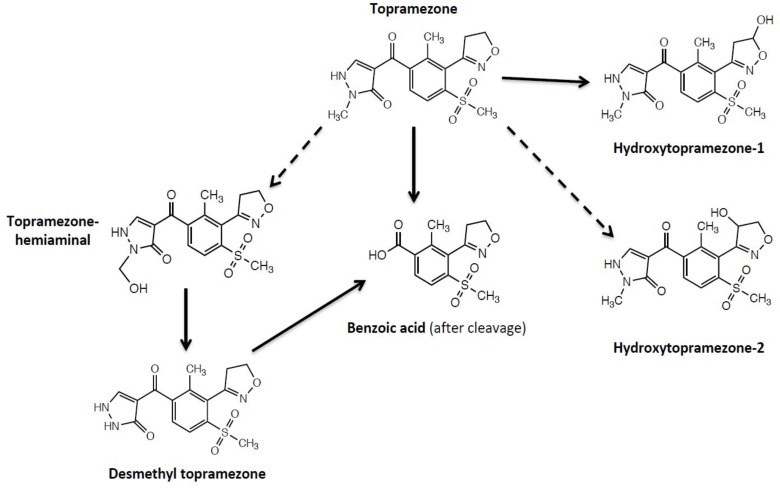
Proposed initial routes of topramezone metabolism in multiple herbicide-resistant waterhemp (SIR population) and tolerant maize. The benzoic acid metabolite of topramezone was only detected in maize, desmethyl-topramezone was a major metabolite formed in maize but minor in SIR, and both hydroxytopramezone metabolites were major metabolites detected in SIR but very low in maize. Dotted lines indicate two possible structures of the hydroxytopramezone-2 metabolite from Figure 7A: the hemiaminal metabolite that likely forms transiently in maize (Kreuz et al., 1996; Grossmann and Ehrhardt, 2007) but possibly accumulates in the SIR population, and a putative isoxazole ring hydroxylation metabolite (positional isomer) that has not been previously reported in plants or animals (United States Environmental Protection Agency, 2005).

Proposed initial routes of topramezone metabolism, based on our current results, in MHR waterhemp (SIR population) and tolerant maize are depicted in Figure 8. The basis for topramezone selectivity in maize is primarily via *N*-demethylation of the pyrazole ring (Grossmann and Ehrhardt, 2007), while mesotrione selectivity in maize (and resistance in waterhemp) proceeds via 4-hydroxylation of the cyclohexanedione ring (Hawkes et al., 2001; Ma et al., 2013). Our findings demonstrate that the SIR population initially metabolizes topramezone by isoxazoline ring/*N-*alkyl hydroxylation at a liable position, indicating a different route for initial topramezone metabolism than tolerant maize (Figure 8), although it is not known if both *N*-demethylation and hydroxylation reactions are catalyzed by the same or different P450 enzymes (Frear et al., 1969; Siminszky, 2006; Grossmann et al., 2011; Hamberger and Bak, 2013; Munro et al., 2013). It is of great interest to determine whether one or multiple P450(s) detoxify the three main commercial HPPD inhibitors applied POST, leading to cross- or multiple-resistance, respectively. In the case of tolerant maize, a single *P450* gene located on chromosome 5 (named *Nsf1* for nicosulfuron tolerance-1; Williams et al., 2006) confers cross tolerance to multiple herbicides within the HPPD-inhibitor family, as well as herbicides from other site-of-action groups (Nordby et al., 2008; Williams and Pataky, 2010).

## Conclusion

Our findings indicate that MHR waterhemp populations possess multiple genes encoding diverse metabolic enzymes that confer complex, herbicide-dependent, cross- or multiple resistance patterns, which may be influenced significantly by prior field-use histories. Potential linkages among the genes conferring HPPD-inhibitor resistance in waterhemp can be explored with segregating F_2_ lines (Huffman et al., 2015) to determine if resistances to mesotrione, tembotrione, and topramezone are actually examples of cross-resistance or multiple resistance (Yu and Powles, 2014). Further mechanistic studies are required to determine whether additional non-target-site resistance mechanisms might be involved in conferring HPPD-inhibitor resistance, in particular reduced cellular transport or whole-plant translocation, since mesotrione is systemic and resistance in waterhemp is a quantitative trait (Huffman et al., 2015; Kohlhase et al., 2018). This can be accomplished by investigating the relative movement of putative metabolically blocked, experimental triketones that are systemic in nature (Beaudegnies et al., 2009). There remains great interest in discovering new chemistries for commercial HPPD-inhibiting herbicides (van Almsick, 2009; Wang et al., 2015; Ndikuryayo et al., 2017; Li et al., 2018). As a result, selection pressures for HPPD-inhibitor resistance will continue to increase in natural weed populations, particularly with the impending commercialization of HPPD-resistant soybean varieties (Siehl et al., 2014), which necessitates novel, integrated management strategies to combat resistance due to metabolic detoxification mechanisms. Given the dissimilar structures and maize selectivity basis between mesotrione or tembotrione and topramezone, further research by our group will continue to investigate physiological mechanisms by which SIR is resistant to the pyrazolone herbicide topramezone relative to triketone chemistry.

## Author Contributions

DR and SK conceived the work. DR designed and supervised the work and wrote the manuscript. AL and AH planned and performed the experiments and generated the data. EM performed the statistical analysis and provided advice on experimental design. DR, JM, and SK analyzed the data and interpreted the results. SK provided the seed. SK and JM revised the manuscript critically.

## Conflict of Interest Statement

The authors declare that the research was conducted in the absence of any commercial or financial relationships that could be construed as a potential conflict of interest.
